# “Using the right tools and addressing the right issue”: A qualitative exploration to support better care for intimate partner violence, brain injury, and mental health

**DOI:** 10.1371/journal.pone.0311852

**Published:** 2024-10-11

**Authors:** Danielle Toccalino, Halina (Lin) Haag, Emily Nalder, Vincy Chan, Amy Moore, Christine M. Wickens, Angela Colantonio

**Affiliations:** 1 Institute of Health Policy, Management and Evaluation, University of Toronto, Toronto, Ontario, Canada; 2 Acquired Brain Injury Research Lab, University of Toronto, Toronto, Ontario, Canada; 3 Lyle S. Hallman Faculty of Social Work, Wilfrid Laurier University, Waterloo, Ontario, Canada; 4 Department of Occupational Science and Occupational Therapy, Temerty Faculty of Medicine University of Toronto, Toronto, Ontario, Canada; 5 Rehabilitation Sciences Institute, Temerty Faculty of Medicine, University of Toronto, Toronto, Ontario, Canada; 6 KITE-Toronto Rehabilitation Institute, University Health Network, Toronto, Ontario, Canada; 7 Dalla Lana School of Public Health, University of Toronto, Toronto, Ontario, Canada; 8 Institute for Mental Health Policy Research, Centre for Addiction and Mental Health, Toronto, Ontario, Canada; 9 Campbell Family Mental Health Research Institute, Centre for Addiction and Mental Health, Toronto, Ontario, Canada; 10 Department of Pharmacology and Toxicology, University of Toronto, Toronto, Ontario, Canada; The University of Texas Health Science Center at Houston, UNITED STATES OF AMERICA

## Abstract

**Background:**

Intimate partner violence (IPV) is a global public health crisis. Often repetitive and occurring over prolonged periods of time, IPV puts survivors at high risk of brain injury (BI). Mental health concerns are highly prevalent both among individuals who have experienced IPV and those who have experienced BI, yet the interrelatedness and complexity of these three challenges when experienced together is poorly understood. This qualitative study explored care provision for IPV survivors with BI (IPV-BI) and mental health concerns from the perspectives of both survivors and providers.

**Methods:**

This qualitative interpretive description study was part of a broader research project exploring employment, mental health, and COVID-19 implications for survivors of IPV-BI. Participants (N = 24), including survivors and service providers, participated in semi-structured group and individual interviews between October 2020 and February 2021. Interviews were recorded, transcribed, and thematically analyzed.

**Findings:**

Four themes were developed from interview findings: 1) identifying BI and mental health as contributing components to survivors’ experiences is critical to getting appropriate care; 2) supporting survivors involves a “toolbox full of strategies” and a flexible approach; 3) connecting and collaborating across sectors is key; and 4) underfunding and systemic barriers hinder access to care. Finally, we share recommendations from participants to better support IPV survivors.

**Conclusions:**

Identifying both BI and mental health concerns among IPV survivors is critical to providing appropriate supports. Survivors of IPV experiencing BI and mental health concerns benefit from a flexible and collaborative approach to care; health and social care systems should be set up to support these collaborative approaches.

## Introduction

Global estimates suggest 26–55% of ever-partnered women have experienced intimate partner violence (IPV) in their lifetime [1–3] and an estimated 10–12% have experienced IPV in the last 12 months [1, 2]. Consisting of behaviours by a current or former intimate partner (e.g., spouse, partner, significant other) that results in “physical, sexual, or psychological harm” [1], IPV is a global public health crisis. In Canada, estimates suggest 44% of ever-partnered women have experienced IPV and 23% have experienced physical violence from an intimate partner [2]. Unlike many other forms of injury, IPV is often repetitive and occurs over extended periods of time, with survivors commonly experiencing multiple forms of abuse (e.g., physical and psychological) [1–3]. There are many concurrent and long-term effects of IPV on survivors, including chronic pain, poorer mental and physical health, and negative impacts on employment [1, 3–9]. One of the often overlooked yet highly common consequences of IPV is brain injury (BI) [10, 11]. Physical impacts to the head or body resulting in an alteration in brain function (e.g., loss or alteration of consciousness, loss of memory) [12], or disruptions in blood flow from strangulation resulting in deprivation of oxygen and nutrients to the brain [13, 14], can cause BIs in survivors. Like IPV, BI can have significant long-term negative impacts on physical and mental health and employment [15–18]. While survivors of IPV are at increased risk for experiencing BI, individuals living with BI and other types of disability are also at increased risk of violence from an intimate partner [19–21]. Therefore, IPV and BI are highly interrelated, complex issues with significant long-term physical, psychological, social, and economic impacts for survivors, their support systems, and the healthcare system [11, 15–18, 22–25].

Poorer mental health is independently associated with both IPV and BI, with elevated risk of anxiety, depression, and post-traumatic stress disorder among both groups [9, 25–28]. IPV-BI survivors have elevated rates and severity of mental health concerns compared to both individuals who have not experienced IPV and IPV survivors without BI [11, 22, 29–33]. Adding to this complexity, characteristics and symptoms of mental health concerns can overlap and interact with those of BI and vice versa [22, 23, 34, 35], which can lead to BI being overlooked [22, 23] and, consequently, going untreated [24, 25]. Not only does this lack of identification hinder access to appropriate healthcare for BI but it can also contribute to reduced effectiveness of mental health treatment [24, 29]. For example, failure to accommodate for challenges that can accompany a BI, such as emotional dysregulation, pain, and cognitive limitations, can reduce the effectiveness of PTSD treatment [23]. Similarly, the physical sequelae of BI such as headache, pain, and fatigue can exacerbate depression symptoms and impact treatment [24]. Conversely, a proactive approach to assessing for and treating mental health challenges in BI can improve clinical outcomes [36].

Given the complexity and interrelatedness of IPV, BI, and mental health challenges alongside the higher use of healthcare services among IPV survivors with BI than those without [37], a better understanding of survivors’ experiences is important for providers in both health and social care sectors to identify and address survivors’ unique needs. A recently conducted scoping review focusing on the interrelatedness of IPV, BI, and mental health challenges identified many articles providing recommendations for service providers, but limited exploration of the impact of these three complex and interrelated experiences on healthcare related needs or service provision [11]. A qualitative approach enables a more nuanced understanding of the complexities of survivors’ lived experience, supporting more informed research, policy, and practice [38, 39]; yet only two of the articles identified in the review explored IPV survivors’ experiences with BI and mental health using qualitative methods. One of these articles reported on findings from another branch of the research project reported here [40]; the other was the only article to explore help-seeking of IPV survivors, focusing on survivors who had experienced strangulation [41]. We therefore used qualitative interpretive description methodology [38, 42] to address this gap in the literature, exploring health-related needs and experiences of IPV survivors with BI and mental health concerns and related care provision/receipt from the perspectives of both survivors and service providers.

## Methods

This study was conducted as one arm of a broader research project investigating employment and mental health among survivors of IPV-BI, with a sub-study exploring implications of COVID-19. Due to the diversity and depth of discussion on these topics, findings are presented in several manuscripts including two published articles, one exploring COVID-19 [40] and one exploring the experiences of BI and mental health among survivors [43], and forthcoming publications exploring the experiences of work and employment among survivors [44]. Findings reported in this manuscript focus on challenges and considerations of providing and obtaining appropriate healthcare and supports for IPV-BI survivors experiencing mental health concerns. This work was grounded in an interpretivist worldview, which understands phenomena as experienced differently by different individuals [45, 46]. Aligned with this worldview, we used interpretive description methodology, which appreciates experiences as both highly individual and encompassing shared realities [38, 39]. This methodological approach is often used in nursing education research because of its ability to appreciate the complexity of experience while developing useful and actionable outputs (e.g., recommendations, training content).

### Participants & sampling

Participants were identified and invited to participate in this study using purposeful sampling via a national Knowledge to Practice (K2P) Network and snowball sampling. The K2P Network includes over 300 diverse community partners, researchers, survivors, and decision makers in the IPV and BI communities from across Canada [47]. Purposive and snowball sampling from this group allowed us to recruit participants who had awareness of or experience with IPV-BI, facilitating richer data on the experiences of IPV, BI, and mental health intersecting. Aligned with the overarching project goals to explore employment and mental health among survivors of IPV-BI, we sought four categories of participants: women survivors of IPV, frontline workers in IPV or BI, upper management (e.g., directors, managers) in IPV or BI, and individuals involved in employment or work-based advocacy (e.g., employers, HR representatives, union representatives). While IPV impacts individuals across the gender spectrum, the majority of survivors are women, informing the focus of this study on women survivors [1, 2, 48, 49]. To be included in the study, individuals needed to be over the age of 18, able to provide informed consent, and able to communicate in English. Survivors did not need formal BI diagnosis to participate. However, we assessed for common indicators of BI in an intake demographic questionnaire and all survivors endorsed experiencing hits or injury to the head, face, or neck and persistent symptoms post injury indicative of BI.

Participants were recruited between October 6, 2020 and February 5, 2021. Study information, including discussion questions and resources in case of emotional distress, were sent to individuals interested in participating. All participants provided written informed consent, returning signed consent forms and demographic questionnaires to the research team via email or letter mail before participating in an interview. Interviews were scheduled via phone or email. A $100 honorarium was provided to survivor participants in the form of a gift card. Research Ethics Board approval was granted by the University of Toronto (protocol #39175) and Wilfrid Laurier University (protocol #6611) prior to commencing this research.

### Data collection & analysis

The first and second author, both cisgender White women pursuing doctoral degrees at the time of interviews one of whom has a BI, co-facilitated semi-structured individual and group interviews between October 2020 and February 2021. The research team included seven cisgender women with varying levels of training (bachelor’s to doctorate), career stages (trainee to senior researcher), and backgrounds (e.g., health services, social work, rehabilitation sciences, mental health, neuroscience). The research questions and interview guides were informed by the first author’s training in health systems and policy research and the second author’s training in social work. Exploration at both a front-line practice and a health system level enabled findings to be applicable at multiple levels of change. Interviews were conducted and audio recorded via Zoom, lasted 60–90 minutes, and covered a range of topics related to employment, mental health and healthcare, and the impacts of the COVID-19 pandemic. Discussion prompts were purposefully broad to encourage participants to share what they deemed most important, enabling us to prioritize participant voices in this work. Given the sensitive and potentially triggering nature of the topics being discussed, participants were provided with a list of resources they could use if they required emotional support. Additionally, one of the interviewers (HLH, a trained social worker) was available during and after each interview to support participants as needed. Finally, participants were encouraged to take breaks, including pausing and resuming on another day if needed.

Interview recordings were transcribed using a professional transcription service and reviewed by the first and second author in an iterative process with data collection to enable adjustment between interviews to further explore emerging topics of interest. The first and second author and a research assistant (AM) conducted preliminary analysis to identify points of discussion related to the three overarching topics of interest for the study: mental health, employment, and COVID-19. The first author thematically analyzed portions of the discussion related to mental health and healthcare within the context of the broader discussion using reflexive thematic analysis as described by Braun and Clarke [50, 51]. This iterative, six-phase process began with re-reading and/or listening to the entirety of each interview to become refamiliarized with the data. After refamiliarization with the data and aligned with the previously conducted preliminary analysis, portions of the interviews related to mental health and healthcare were compiled for further coding. Working with the mental health and healthcare focused portions of the interviews, codes were assigned inductively based on impressions from previous read throughs and deductively based on the research questions. During the refamiliarization and initial coding phases, survivor transcripts were reviewed first, to center and prioritize this often-silenced group. These codes were then grouped into themes, loosely based on the two research questions guiding this arm of the research: what are the BI and mental health experiences of IPV survivors, and what are the challenges and considerations of providing and obtaining appropriate healthcare and supports. Themes were clarified and refined with the research team in an iterative process as the two reports, one focused on each research question, were written. Braun and Clarke’s guidance on thematic analysis evaluation informed the reporting in this manuscript [51].

## Findings

Individual (n = 19) and group (n = 2) interviews were conducted with 24 participants including 6 women survivors of IPV, 7 frontline workers, 5 executive directors/program managers, and 6 employers or human resource/union representatives. Most participants identified as cisgender women (96%) with European ancestry (71%). Four participants identified as Indigenous (8%) or as Black or of African origin (8%); three participants identified as South Asian (4%), Middle Eastern (4%), or of multiple visible minorities (4%). Service providers, including frontline workers and upper management, had worked in the IPV sector for an average of 13.8±11.3 years and worked in organizations such as victim services, shelters, BI services, and advocacy. As noted in Participants & Sampling, all survivors had experienced at least one injury to the head, face, or neck from IPV-related violence, with most (83%) experiencing loss or alteration in consciousness and all endorsing 10 or more lasting challenges that are indicative of a probable BI. Four themes were developed around the challenges and considerations of providing and obtaining appropriate healthcare and supports, including: identifying BI and mental health components among IPV survivors, a toolbox full of strategies, connecting and collaborating across sectors, and underfunding and systemic barriers hindering access. In our final findings section, recommendations provided by survivors and service providers during the interviews are synthesized and presented in Table 1. Supporting quotations are embedded in the text with the participant identified by their group (e.g., survivor, frontline worker) and their participant number (e.g., P1) or their focus group number if they participated in a group interview (e.g., FG1). To be true to the participant’s voice, we have maintained their terminology for BI and IPV in the quotation material (i.e., BI or brain injury, IPV or intimate partner violence) despite using acronyms in the body of the text.

**Table 1:** Survivors’ and service providers’ recommendations to support better care for intimate partner violence (IPV), brain injury (BI), and mental health.

*Recommendations*	*Supportive Quotations*
***Identifying BI and mental health as contributing components***	
Consider BI in IPV survivors–be aware of BI signs and symptoms and look for them in the individuals you serve	“When women come in with mood dysregulation, when women come in with significant depression or anxiety, are you thinking intimate partner violence there? Are you thinking that maybe this is an injury to the brain that could be factoring in? And I just don’t think that’s where people go. I think people go to trauma and the effects of trauma on the brain and this has been a huge area in IPV, we all took a million different workshops on it. They’re not actually thinking your brain has actually received physical damage. So, I do think that that would be transformative”–Manager (P8)
	“The key message that frontline workers need to hold about survivors with acquired brain injuries, I think just being aware that that is a possibility that a woman might be experiencing that is super important. I think a lot of people it doesn’t even enter into their mind, and so there’s, I think, a lot of frustration or misunderstanding that could be avoided if you entertained that that is potentially what is happening or what someone might be experiencing. And then being equipped with some tools to support someone who might have an acquired brain injury.”–Frontline Worker (P9)
Build space into interactions to allow survivors to process and formulate a response	“Like she can’t have nurses coming in and hammering questions at her and then, [she’s] unable to even process the first question before they ask her 20 other questions. And then get mad at her because her response is to get mad and tell them to stop. And then it turns into this whole other thing. Because now all of a sudden the nurses are thinking that she’s being aggressive when literally she just needs everybody to stop talking for like 30 seconds. But that’s just such a–like an unheard-of tactic.”–Frontline Worker (FG1)
***A toolbox full of strategies and a flexible approach***	
Focus on what the survivor needs from their perspective–be willing to support the survivor in the way they need (or find appropriate supports if outside of your scope)	“Some workers show up, they come to work with [the perspective] ‘I’m here to fix these broken women. I have the knowledge and expertise … and so I’m here to fix these women.’ And then when they meet a woman who says very politely, ‘I do not need fixing from you thank you, I need housing, can you help me fill out my form. I need childcare, I need job training. . . I need to file a motion in court–I have a specific need.”–Executive Director (P6)
	“There’s a lot of [treatments and supports] that are helpful, but women don’t know about them, because it’s not–it’s sort of trial and error, what works for some person, might not work for another person.”–Survivor (P4)
	“I don’t think they understood how difficult things were for me [because of my TBI], but they got it, you know because I would go in to the office and I knew that step by step it was OK, who did you tell me to call? What did you tell me to do? And they’d be OK we told you to [call this person] and I’d have to write it down or put it on my phone and I had a screen lock on my phone so he couldn’t see it. And I had things stuffed in my car. You know it was just, you know it was like OK where did I put that? Where did I put that note? And so I had to have cues to remind myself what did I do with certain things?”–Survivor (P11)
You cannot put a timeline on recovery–be aware that healing after BI is not a linear process	“One piece that didn’t come up that I wanted to share that was a challenge and continues to be–and it piggybacks on the conversation around the need for long-term services. And that’s we just can’t put a timeline on recovery sometimes–most times, is how hard it was to try and serve women in the Transition House in 30 days.”–Program Coordinator (P10)
***Connecting and collaborating across sectors***	
Increase understanding and awareness of possible concerns and relevant supports	“But [my BI worker] literally, for weeks, would sit there with me and explain why I was feeling certain ways, workarounds for doing specific things, like, really basic, basic tasks like getting post-it notes and sticking them everywhere. And I lived like that for almost a year and a half, with post-it notes everywhere, and books, keeping track of everything … but I learned a lot. And I think the level of care and outreach from her enabled me to think more positively about what my future could be.”–Survivor (P5)
Build your own networks for support and referral	“I feel like the informal connections between teammates is crucial, especially those working in separate sectors. … I think those informal connections are really, really key. Just a call and say … we just wanted to check in. I wanted to talk about CD or AA or whatever. And I wanted to let you know that this is one of the things that we worked on recently … it’s not a burden to ask. It’s not a burden to share. And so, it’s in the best interest of the client. How can we just have this conversation? How can we just move this forward, without it being so formal? … I think the informality part is–I’m finding has been really beneficial and useful … of course privacy, confidentiality all that stuff is super important. Keeping and files and documents and names and all of that, that is definitely important. But I think just, yeah, making those connections and feeling connected enough in your own community.”–Program Coordinator (P10)
Support cross-sectoral connections and wayfinding via navigational and/or peer support	“There needs to be an accessible resource base that survivors can access. That has the advocates that has the knowledge of where the resources are that the survivor can go to.”–Survivor (P13)
	“We need peer support, but we do not value it at all enough. We don’t recognize experience as an asset. We don’t recognize experience as knowledge.”–Survivor (P1)
	“You know, to link it, because it’s hard enough when you have a brain injury, to try and find one right person to help you, when you are constantly looking for other. So it would be so nice to have it all linked.”–Survivor (P5)
***Underfunding and systemic barriers***	
Reduce barriers to access mental health supports (reduce criteria and increase funding)	“I think it would be great if well, firstly there was a little more accessibility for women to access women’s health services. There’s currently of course wait lists and all the rest of it and you have to be, you know, we are underfunded, you know, we’ve had women for example who are suicidal and we convince them to call 911 and they spend two hours in the emergency room, the mental health emergency room, and then they’re sent home or sent back to the transition house and you’re like wait a minute … So, there’s certain huge barriers and this is, mental health is hugely underfunded.”–Manager (P8)
Make BI supports accessible without a diagnosis.	“I think that, for me, it would be more brain injury support for women who aren’t diagnosed and don’t necessarily fit the category medically, but have had brain injury because of the partner violence, and sort of getting that piece of support for them from that perspective without having to exactly fit the box.”–Frontline Worker (FG1)
Consider using screening to support access to BI supports	“If formal diagnoses are difficult to obtain, is there other supporting documentation that an individual could acquire that would support? Like if a social worker is trained to recognize the signs of a traumatic or acquired brain injury, not that we could diagnose, but we can offer a support letter indicating like ‘based on my experience, it is my belief that this client may be experiencing the impacts [of BI]’”–Frontline worker (P9)
Financial support for services not covered by insurance.	“But some women … they don’t have the money, so they see the price and they just you know drop it right there. But it’s the same with psychiatric services, I mean all they do now is give you pills, they don’t give you any kind of counselling. So counselling is–unless you can get into a free service, which is inundated with women–then you’re paying, and it can be upwards of $125 for an hour.”–Survivor (P4)
Increase awareness of healthy relationships	“We don’t teach girls and boys just about our bodies and what you can accept from someone else or not in terms of space and competence. And all those boundaries and issues, like we don’t–nobody’s got a clue.”–Survivor (P1)

### Identifying BI and mental health as contributing components to survivors’ experiences is critical to getting appropriate care

Participants across groups emphasized the importance of identifying both BI and mental health challenges as components contributing to IPV survivors’ experiences while acknowledging it is difficult to do so. As discussed in a companion paper exploring experiences with BI and mental health in IPV [43], these experiences meld into “a whole big ball of all-togetherness” that was “hard to pick apart” (Survivor P1). When exploring the impact of that ‘all-togetherness’ on care provision, service providers noted that “It’s really hard to tease out what’s the important or what’s the key piece … because of the complexity” (Frontline FG1). Despite this complexity, survivors and service providers alike recognized that identifying these components is essential to getting survivors appropriate care.

*“I just wonder how often we’re maybe not helping our clients because we’re like ‘oh there’s a mental health issue here’ and we’re using all our mental health tools and then, but it’s not a mental–like it’s impacting a person’s mental health but we’re not equipping them with the right support, so we’re like having a talk therapy group about something and they’re like ‘I actually just need a way to remember certain things’ or like, I don’t know, I just wonder if we’re like spinning our wheels because we’re not actually using the right tools and addressing the right issue.”–Frontline worker (P9)*

It was also recognized that when there are such complex interactions of health issues, some interventions may not be as effective until other components are dealt with.

*“There’s one woman … we had her in and we’re filling out all these forms and we’re trying to get housing and I’m talking to her Addictions Outreach worker, and at the end of the day, none of the housing and BI strategies are going to work until she’s sober.”–Program Coordinator (P10)*

*“I said that I had polymyalgia and I wanted to know if [the neurologist] knew anything more about it*. *And she actually is the one that said that the neurofeedback wouldn’t work until the polymyalgia rheumatica was under control and that was why I wasn’t seeing much benefit from it*.*”–Survivor (P4)*

While it was acknowledged that it might not be possible to determine the root cause of a symptom or health issue, service providers spoke about the value for survivors in knowing that BI might be impacting their experience.

*“It’s so important for people to have the opportunity for someone to tell them, hey, this might be what’s going on for you, so maybe you can feel less “crazy” you know–there’s an explanation for what you’re feeling. There’s an explanation for why you’re here. You didn’t do anything wrong. And I think people need to hear that, because otherwise there’s so much self-blame and confusion. And then you don’t know how to help yourself, because you don’t know what’s going on.”–Frontline worker (P7)*

Communication challenges that can arise after BI were discussed as relevant across the care continuum, including but not limited to difficulty processing rapid-fire questions in a healthcare environment and needing extra time to formulate and communicate their thoughts.

*“I think a big underlying factor is IPV TBI [traumatic BI] sufferers and survivors have a hard time communicating, they have a hard time getting their story out, getting their thoughts in order. They need to have time to gather their thoughts before they can talk. Sometimes it takes me a while to get my words out, even now. So that’s kind of the overlying thing is that if we’re not able to quickly think, don’t judge us because we’re not. I find there’s a lot of judging that goes on. We can be called stupid, we can be called dumb, we can be called lazy. But in reality, we can be researchers, PhDs, doctors, we can be–you know, we can do anything that we want to do. It’s just we had an injury that does affect us every day at every second, but we live with it. We’ve learned to cope with it. So just allow us the courtesy of being equal to you, who are lucky enough not to have an injury.”–Survivor (P11)*

Without the recognition and/or diagnosis of BI, survivors are left without an understanding of the impacts a BI might be having on their lives and are prevented from accessing supports that might help them to navigate and mitigate those impacts. Service providers working with survivors of IPV should consider both mental health concerns and BI as possibilities and take that into consideration in treatment and support planning (Table 1).

### Supporting survivors involves a “toolbox full of strategies” and a flexible approach

Identifying the components playing a role in a survivors’ experience was noted as a critical first step to being able to appropriately support survivors. Service providers recognized that care plans for IPV survivors with BI and mental health concerns need to be flexible. Distinguishing between a symptom originating from BI versus from mental health might not be possible, but a flexible approach to care can help counteract this challenge.

*“If it turns out that the reason why you’re not remembering things isn’t your BI, it’s because you are intoxicated, then we have–then we want to start at a different place … sometimes I have a hard time sourcing where the challenge is coming from. And the only thing I found to counteract that is just to try things… I’ve got a toolbox full of strategies that we can try and implement and see if it makes a difference for you. And if it doesn’t, then we’ll try something else”–Program Coordinator (P10)*

Recognizing IPV, BI, and mental health are all likely contributing to a survivors’ experience means a provider’s toolkit can expand to include not only IPV, BI, or mental health strategies, but all three. A key part of this flexible approach, recognized by survivors and service providers alike, was tailoring the care to the individual’s specific needs. Participants across categories challenged service providers to ask: “what does the survivor in front of me need right now?” and have that conversation with the survivor. Sometimes, this requires identifying the correct professional to assist, but other times more practical supports are key first steps, including working with the survivor to determine how best to support them.

*“When something’s happening where a woman is struggling, OK well what–where do we start to try to begin to address this or get things back on track? Do we need to call the psychiatrist? Do we need to call the neurologist? Do we need to call the counsellor? Do we need to just come in here with some practical supports and go yeah, we’ll clean your bathroom, and we can make you some lasagnas? It can be all of those things … I think for each individual woman that you have to kind of figure out what is the most helpful for her.”–Manager (P8)*

The need for flexibility also extended to timelines, particularly in the journey of healing from BI. As discussed in the companion paper [43], the impact of IPV “doesn’t just end” (Survivor P2). When considering care provision for survivors, particularly survivors with BI, this lasting impact can include a potentially rocky healing journey with a timeline that cannot be pre-determined.

*“And I think from what we’ve learned from [the BI] team is a lot about timeline and the patience that it takes just the day to day and just the expectation that our BI folks have that they just expect that it’s going to be a very rocky road with lots of setbacks. And I think we’ve learned a lot about that trying to–just alter our expectations a little bit and not become feeling like oh, we’ve put all this work into it and now it’s getting derailed … I think we have had that feeling sometimes like well, why is she sabotaging all the stuff we’ve been working on? And not recognizing that there’s a brain injury there that really is a huge component of that.”–Manager (P8)*

With each survivor experiencing IPV, BI, and mental health differently, with unique family and social circumstances, service providers are encouraged to focus on what the survivor needs from the survivor’s perspective and to recognize that you cannot put a timeline on recovery (Table 1).

### Connecting and collaborating across sectors is key

Recognizing the importance of a toolbox that includes IPV, BI, and mental health strategies requires also recognizing that one provider is likely not fully equipped in all areas; this is where connections and collaborations across sectors is key. Knowledge of and collaboration with providers across sectors was highly valued across participants. Survivors who were connected to BI associations spoke about them as critical to their own healing journey. In one example, the survivor’s family doctor recognized there was something wrong but did not know how to help her, so he facilitated a connection with their local BI association. This connection was pivotal to the survivor’s healing journey and helped further connect her with necessary supports.

*“And the brain injury association, I managed to find them through my family doctor, and they put me back together. Literally, they put me back together. They sent out an outreach worker, who worked with me for almost two years and arranged for my physio … they arranged [occupational therapy] and did testing for that and arranged for the neurological testing at the hospital. And they literally saved me, because I was lost, I really was lost.”–Survivor (P5)*

The survivor further spoke about the value of having someone on her care team who understood BI and could support her in navigating that experience.

Connections between BI associations and IPV supports were also noted by service providers and survivors alike as being incredibly valuable. For example, a survivor shared that the collaboration between the BI association and a local women’s shelter helped the shelter understand her challenges with memory and planning, which allowed them to better support her when she was leaving her relationship.

*“I had a counsellor with the brain injury association who witnessed some of the abuse. She put me in contact with the local women’s shelter … and we worked with the women’s shelter in getting me an apartment, in getting a safety plan and everything set up.”–Survivor (P11)*

Service providers echoed this, with some sharing their own experiences collaborating across traditionally siloed domains to support survivors and improve awareness of the circumstances and symptoms often accompanying BI.

*“When she goes into her supportive housing, [we’re going to have] a case conference so that we can have me come in with the brain injury perspective, [the mental health case worker] is going to come in with the addictions perspective. So that we can make sure that when she goes into this housing that they understand what the realities of her situation are.”–Frontline worker (FG 1)*

Most of the service providers we spoke with, though they worked with IPV-BI survivors, worked predominantly in either IPV or BI. Notably, service providers in both fields were very aware of mental health concerns–IPV service providers were often trained mental health professionals (e.g., social workers) while BI service providers knew both how to recognize signs and symptoms of mental health challenges often encountered among individuals with BI and to refer out to relevant supports. Experts in their respective fields, these service providers spoke about how valuable it was working in a collaborative environment with experts in other areas.

*“I work in IPV so, I personally find myself still very short on the expertise of the brain injury side of things. And so, it’s really helpful to have my clients plugged in with the brain injury professionals … Coming from a place where we assume everything is just psychological trauma, to recognize that, oh my gosh, there’s something much more here, has been quite eye opening.”–Frontline worker (FG1)*

*“I think coming from the BI side of stuff*, *it was awesome to see the perspective that the IPV workers come from*, *because they’re completely different from where we kind of come from in the BI stuff … when you’re working with people specifically focused on brain injury*, *a big part of that is managing mental health*, *managing substance misuse*, *managing unhealthy relationships and potential for violence*, *either from the partner or from the individual*, *because emotional regulation is a really big piece of BI*.*”–Frontline worker (FG1)*

Service providers in management positions recognized both pieces as critical to appropriate care. Cross-training between fields, while “super important for the recognition and awareness piece,” cannot make one individual a jack-of-all trades and that, they would argue, should not be the goal. The value of connections and collaborations between disciplines or professions is in being able to identify challenges related to BI, IPV, or mental health and work with colleagues to better support the survivor in a wholistic and person-centred way, connecting the survivor to appropriate supports, if necessary.

*“The women that we’re working with would still need both … So, it was less of, how do we train IPV workers in BI–it was more of, how do we ensure everybody can recognize and see that this might be a BI issue, or this might be a trauma issue, or we might be looking at something completely different, like addictions, or mental health, or various other things”–Program Coordinator (P10)*

Beyond the IPV and BI specific spheres of expertise, service providers and survivors emphasized the need for connection with other forms of support. Connection to and relationships with Elders was noted as an important aspect of culturally relevant and inclusive supports for Indigenous survivors.

*“The services I received from them were from visiting Elders who came in and did counselling and support. And they were far more supportive than anybody I’ve found to date. Because they were there to talk when you needed it. And they were able to give direction and they have healing modalities.”–Survivor (P13)*

*“I think we always try … to refer more to cultural supports and be a little more creative with that*, *and certainly more cognizant of*, *you know*, *maybe it’s not a counsellor who’s doing*, *you know*, *[cognitive behavioural] therapy*, *but you know*, *access more of our Elders in our communities*. *But the challenge too is again*, *you could have different Nations who come here and that*, *you know*, *the Anishinaabe Elder may not be the most appropriate–so we do rely on a lot of networking up north*.*”–Executive Director (P3)*

Survivors emphasized the value of someone to help navigate the different sectors and care providers they might need to access across their healing journey—someone dedicated to supporting the survivor as an individual, who might themselves be a survivor.

*“I know for instance like in homicide cases right, OK, so, it’s a serious, serious thing obviously, it’s like a big, big crazy bad example, but honestly like that’s the one time ever where there’s one person like a volunteer care support person who’s basically assigned to you, and they go with you … and they are just there for you. They are just there to be your navigator, advocate, whatever you call that, and you know, how amazing would it be if everybody had like one of those”–Survivor (P1)*

Many different sectors and professionals are involved in a survivor’s healing journey. Having an increased understanding and awareness of possible concerns experienced by IPV survivors, such as BI and mental health challenges, and being aware of and able to refer to relevant supports is critical for any service provider interacting with survivors. This could involve building and maintaining networks for support and referral and maintaining a repository of resources. The assistance of (peer) navigators could support survivors in navigating the system (Table 1).

### Underfunding and systemic barriers hinder access to care

Across the interviews, participants spoke about the challenges IPV survivors face in accessing care. For mental health, both high demand and strict requirements were barriers to accessing services. Survivors and providers spoke about struggles with healthcare and support organizations being understaffed and overworked. For survivors, this translated to long wait times, particularly for mental health services, which was also noted as a barrier to accessing care.

*“As far as counselling and like the sexual assault crisis centre, I mean they’re so overloaded, which is hard for anybody that’s coming out of the system … if you need somebody, you need somebody then. And with some people if you don’t get them in then, the impetus is gone, you have to wait six weeks. Then they–ah everything’s fine, I don’t need to go, you know what I mean. So, I think there needs to be some change maybe in the way they do some of the counselling services.”–Survivor (P4)*

In some cases, survivors were bounced from organization to organization with services too overwhelmed to even have a waitlist.

*“My doctor did nothing to get me into counselling. I went on my own … they said they were so busy they couldn’t even put me on a waiting list … I called [other organizations] and I was told they couldn’t help me.”—Survivor (P13)*

For service providers working in understaffed environments, being overworked led to priorities shifting, resulting in sub-optimal care.

*“I think, like, so many people are just so overwhelmed that the questions just don’t get asked, people just don’t care, we need to check this box. And that happened for me at my job, you know; I had some concerns about how I was working, and the motto at the time was just, they’re safe, it’s okay. They’re safe, it’s okay. But no, I want to do more than just offer a building where people are safe.”–Frontline worker (P7)*

This not only impacted the care survivors received but also had negative impacts on service providers who wanted to do more but did not have the capacity. In some instances, this lack of capacity meant service providers were doing the ‘bare minimum’ because that was all they had time for. In other instances, being stretched thin led to tension and conflict between service providers and survivors.

*“Also just staffing capacity in general, because I think organisations under staff routinely, which regardless of how well-trained you are, if your organisation is understaffed you will never have the capacity to support someone who’s like incredibly emotionally dysregulated while you’re supposed to be serving lunch and running the front desk. It’s just like an unrealistic expectation, and all too often. And, again, that’s funding, and maybe larger things, but I think it’s an incredible irresponsibility on management of organisations to understaff the very frontline workers.”–Frontline worker (P9)*

In some instances, program requirements prevented survivors from accessing care when they most needed it.

*“I’ve had people that I’ve worked with who are dying to get–like figuratively and literally dying to get into detox so that they can go to rehab. And they get told, OK, well you have to wait two weeks, you can’t come in sober, so the expectation is they’re going to continue to use for two weeks, even though they have this drive to get clean. And you know I’ve had people that I work with show up at the hospital and try and overdose in front of a nurse because it was the only way that somebody would take her seriously.”–Frontline worker (FG1)*

Strict program requirements were also a challenge encountered with BI supports. Providers knowledgeable about BI services noted that many require formal diagnoses, which may not be accessible for survivors and consequently be a barrier to accessing support.

*“If you don’t have all the scans … to prove this is where your brain injury is then you don’t get the support around the brain injury … that’s just not realistic for women who have experienced intimate partner violence. And I think that’s why it gets framed often as a mental health crisis, right? Because that’s something you can get help for because you don’t need a brain scan to be diagnosed … I think it’s easier to apply [a mental health] label for someone who’s maybe two, three years post brain injury and has never, ever gone to an emergency room.”–Program Coordinator (P8)*

Formal BI diagnosis is required to access many medical BI supports. Service providers suggested reducing criteria for both BI and mental health supports and increasing funding for these programs to enable better access. They further suggested allowing other supporting documentation (e.g., screening) to facilitate access to BI supports (Table 1).

Financial concerns were also noted as a challenge for survivors. Not only are significant costs incurred when seeking health services that are not covered under provincial/territorial health insurance plans, but there are also costs associated with creating the space to heal, be that time away from work, support for childcare, or other supports.

*“You might try it, you know, you don’t know how much time to give it, because it’s expensive. I mean, I’d probably be rich if I hadn’t spent so much on healthcare. But I wanted to be well. I wanted to enjoy my retirement.”–Survivor (P4)*

*“There’s a minority of the women at least that I work with that at least have access to economic supports that could allow them to take some time off to recover at least a bit from this concussion or at least kind of have some accommodations specific to that but the vast majority of women I work with do not have access to those kinds of supports*.*”–Service provider (P14)*

Finally, there were barriers related to the relationships themselves. Survivors and providers noted the challenge for many survivors with recognizing what is not ‘normal’ both in their relationship and with their mental health and cognition. Without first identifying that something is not right, knowing to seek care or what and where to seek it is a challenge.

*“Yes, barriers are language. Barriers are, again, kids. [Laughs] the kids, ugh. Barrier is, again, stigma, and a barrier is psycho-ed. So many folks don’t even know that they’re dealing with PTSD. So many folks don’t even know that it’s not okay for your husband to call you a bitch every day. It feels like suffering is normalised almost.”–Frontline worker (P7)*

However, survivors recognized that even after acknowledging something was not right, seeking help could increase their risk of violence.

*“I started counselling probably maybe the second, third, maybe third year that I was in my relationship. And it was not received very well … there were violent incidents simply because I was going to counselling.”–Survivor (P2)*

While there are many challenges facing survivors accessing care, survivors and service providers identified several opportunities to combat those challenges, including building space for communication, reducing barriers to accessing BI and mental health supports, and increasing funding and psychoeducation to support survivors (Table 1)

### Recommendations from survivors and providers

Many recommendations from survivors and service providers to support better care were made across interviews; these have been noted throughout the findings sections above and compiled in Table 1 with additional supporting quotations. Implications and contextualization of these recommendations are provided in the Discussion.

## Discussion

Four key themes around the challenges and considerations to care provision for survivors of IPV with BI and mental health concerns were developed from individual and group interviews with survivors and service providers. Firstly, survivors and providers alike recognized the importance of identifying both BI and mental health concerns among IPV survivors. Though often challenging to identify, knowing that all three pieces played a role in the survivors’ experience was critical to developing a plan for support and treatment. This led into the second theme, that supporting survivors involves a “toolbox full of strategies” and a flexible approach. As distinguishing between symptoms resulting from BI versus IPV versus mental health is challenging at best, participants across categories spoke about the need to try different things in order to find a solution that works for the individual, tailoring care plans to their specific needs. The interrelatedness of BI and mental health, and the complexity arising from that relationship, were recognized as needing support from a variety of different professionals; this underscored our third theme, the need to connect and collaborate across sectors. Service providers and survivors alike spoke to the benefit of experts in BI, IPV, and mental health collaborating, noting that collaboration was preferable to trying to make one person an expert of all three. Finally, survivors and service providers noted that underfunding and systemic barriers are a significant hinderance to accessing appropriate care, with implications ranging from long wait times to strict access requirements to financial barriers.

Recommendations from survivors and providers were provided throughout and summarized in Table 1. Most notably, survivors and service providers recommend that anyone supporting someone who has experienced IPV should be aware that they might have experienced a BI and consider that possibility in their treatment and support planning. The implementation of the CARE (Connect, Acknowledge, Respond, and Evaluate) advocacy framework is one example of how increasing awareness and providing supportive tools for recognizing BI and mental health concerns among IPV survivors can support better care [52–54]. This framework has been adopted across a network of IPV service organizations, increasing awareness among staff and leading to structural and functional support provision for IPV survivors that took BI into account. An important part of providing appropriate support includes having flexible approaches to care that meet survivors’ unique needs as the survivors themselves see them. A flexible approach requires looking at the totality of what an individual is experiencing and having strategies or supports addressing concerns from a variety of perspectives. For example, a service provider recognizing communication, processing, or executive function challenges as potentially related to an unaddressed BI could lead them to adjust the setting and supports around appointments (e.g., quieter space, more time for the appointment, providing appointment reminders when safe to do so) rather than assuming the survivor is difficult or unmotivated [53]. Recognizing the involvement of a possible BI in a survivors’ experience might also prompt a shift in approach for their mental health treatments, leading to more successful mental health treatment and possibly also improving BI symptoms, as noted in the introduction [23, 24, 36]. Recommendations for service providers (and their organizations) to develop networks for support and referral. Connections between IPV-focused organizations such as shelters and second-stage housing organizations, mental health care providers, BI associations, and local community health organizations could help ensure survivors receive (or can be referred to) appropriate supports regardless of their first point of access.

Survivors and service providers noted a need for flexibility and an understanding of complexity, a need that is echoed in the bodies of literature focusing on subsets of the three components discussed here (i.e., IPV, BI, and mental health). A 2020 study exploring mental health care for IPV survivors emphasized the need for flexibility when providing evidence-based treatments in this context [55]. The findings explored improved coordination between therapeutic and advocacy services as well as respect for the complexity of clients’ lives and support for self-determination as key elements to effective, trauma-informed, and people-centred care [55]. Similarly, a recent systematic review highlighted the need for coordination and collaboration in care for individuals with BI and mental health concerns in part to support that flexibility of approach [56]. The review used the World Health Organizations’ five strategies for integrated people-centred health services as a framework, which includes “engaging and empowering people and communities” and “coordinating services within and across sectors” [57], both which came through strongly in the findings and recommendations arising from this study. In order to treat and refer effectively, service providers across sectors need to be able to recognize the diverse contributors to IPV survivors’ health service needs, understand how these factors may interact and be able to collaborate with other professions and sectors to provide care.

While collaboration was recognized as being invaluable, previous work has identified bureaucracy and highly formalized processes of engagement as barriers to forming those connections [10]. Many of the examples of collaborative care shared in this work were a result of service providers making those informal connections “off the side of [their] desks.” The family doctor who made the connection to the local BI association, the BI worker who collaborated with the shelter to provide BI sensitive supports, and the BI worker collaborating with the mental health provider to provide context to supportive housing were all cases of someone making the right connection to get the survivor the support they needed. This is often the provider going above and beyond. Survivors who are not fortunate enough to be supported by someone with the capacity or inclination to take that extra step are left without that care. Inclusive, collaborative, and financially supported connections between service providers would greatly benefit survivors. Ample support for building trusting, respectful, collaborative relationships is needed to bridge and integrate the diversity and complexity of different settings (e.g., acute and community-based care), organizations (e.g., hospitals, community health centres, mental health agencies), and professions (e.g., social workers, physicians, nurses) involved in interdisciplinary, collaborative care teams [58].

Finally, a noted barrier for accessing BI related services was the need for a formal diagnosis. There are many challenges associated with diagnosing BI, including injuries classified as “mild” (i.e., shorter duration of loss of consciousness or post-traumatic amnesia) often not presenting on many diagnostic scans used to identify more severe injuries [59, 60]. Therefore, in many populations where access to imaging might be more challenging, including IPV survivors, the use of screening tools to identify probable BI is considered the agreeable standard [61]. A variety of screening tools have been used to identify BI among IPV survivors, including a modified version of the HELPS screening tool [48, 62], Brain Injury Severity Assessment [63–65], and Veterans Affairs TBI screening tool [29, 33, 37], among others. A specialized module of the Brain Injury Screening Questionnaire tailored to and validated in IPV survivors has also recently been developed, which is a promising step for the field both in service provision and research [66]. Service providers, particularly those trained in screening or identification of probable mental health concerns, spoke of a willingness to be involved in similar screening processes for BI, particularly if that screening could support survivors’ access to required supports in the absence of a formal diagnosis. Incorporating the use of validated measures into service provision, where appropriate, and expanding inclusion criteria to allow for individuals with positive screens to access more formalized BI supports could be a promising step towards getting IPV survivors appropriate BI care.

### Strengths, limitations, and future work

This work is the first, to our knowledge, to explore service provision for IPV survivors experiencing BI and mental health concerns from the perspectives of both survivors and service providers. Including both survivor and service provider perspectives enables a more holistic understanding of service provision to better support survivor-centred care. The qualitative nature of this work allowed for in-depth exploration into the experiences of the survivors and service providers interviewed. While their experiences do not encompass all the ways in which IPV, BI, and mental health can be experienced, the findings and recommendations identified through this work are broadly applicable to service providers supporting IPV survivors with BI and mental health concerns [67]. This work also provides valuable insight into care provision in a publicly funded healthcare context. The Canadian healthcare system covers medically necessary health services including “hospital services, physician services, and surgical-dental services” [68]. Most of the literature exploring mental health and BI in IPV survivors is based out of the United States, where healthcare is largely funded by private insurance carriers [11]. While we had participants from across the country working or receiving care in settings ranging from rural communities to large urban centres, we did not have enough representation to truly compare and contrast experiences in different settings. Future exploration into experiences specific to different contexts is necessary to inform appropriately tailored care pathways.

As this study was part of a multi-pronged project, interview guides additionally included questions related to employment and COVID-19. While this allowed us to explore connections between these three topics (mental health, employment, and COVID-19), it may have limited the depth of discussion possible for mental health and healthcare-related experiences. Additionally, study recruitment and interviewing occurred in the early stages of the COVID-19 pandemic, when all outreach and interviewing needed to be done virtually (e.g., email, phone, videoconferencing). The modality of outreach along with the general turmoil of living through a pandemic may have impacted who we were able to reach and who was able to participate in the study. The requirement to interview via videoconferencing had additional benefits and potential drawbacks for this study. It allowed us to interview individuals from across Canada, which would not have been feasible if interviewing in person. Conversely, the virtual modality may have hindered rapport building with participants and the flow of conversation during the interviews themselves. A full discussion on the impact of COVID-19 on survivors and service providers as identified through this work is published elsewhere [40].

We focused this work on women as they represent the majority of IPV survivors; however, rates of IPV are higher, proportionally, amongst sexual minorities and trans and gender diverse persons than they are amongst heterosexual cisgender individuals [69–72]. To appropriately design social and health care supports that are welcoming and supportive for all survivors, more community engaged research with the trans, gender diverse, and queer communities is required. In noting this, we recognize that there are many barriers unique to these communities hindering access to care. For example, a recent systematic review identified numerous psychological, sociological, and legal barriers to lesbians experiencing IPV, including the heterosexist frames of reference of many institutions, most notably the erasure of violence in same-sex relationships, particularly relationships between two women [73]. For trans and gender diverse individuals, transphobia, misgendering, and needing to educate providers on trans-inclusive language and practices are notable barriers to seeking and accessing supports [74–78]. We are aware of ongoing work aiming to improve care for trans and gender diverse survivors of IPV through the development of intersectoral networks and trainings for service providers [79–82].

Finally, this research did not explicitly explore the impact of race or ethnicity on care provision. Racism in healthcare is a critical challenge facing our health systems and impacting the support IPV survivors and individuals with BI experience [83–85]. Future dedicated exploration of healthcare experiences among Black, Indigenous, and other racialized IPV survivors with BI and mental health concerns, as well as exploration of the impacts of other social determinants of health with more diverse participants is needed.

## Implications & conclusions

This research contributes to the small but growing body of literature exploring BI and mental health concerns among IPV survivors, adding much needed context from a publicly funded healthcare system. IPV survivors with BI and mental health concerns have unique care needs that must be considered by service providers in healthcare and beyond. Identifying BI and mental health as health concerns contributing to IPV survivors’ experiences and using that information to develop flexible care plans with collaboration across disciplines is critical to appropriately supporting survivors.
